# Reflection on the teaching of student-centred formative assessment in medical curricula: an investigation from the perspective of medical students

**DOI:** 10.1186/s12909-023-04110-w

**Published:** 2023-03-02

**Authors:** Tianjiao Ma, Yin Li, Hua Yuan, Feng Li, Shujuan Yang, Yongzhi Zhan, Jiannan Yao, Dongmei Mu

**Affiliations:** 1 School of Nursing, Jilin University, Changchun, Jilin Province China; 2 School of Public Health, Jilin University, Changchun, Jilin Province China; 3 Division of Clinical Research, The First Hospital of Jilin University, Changchun, Jilin Province China; 4 Institute of Communication and Social Governance, Jilin University, China Changchun, Jilin Province

**Keywords:** Formative assessment, Medical students, Student-centred, Student satisfaction, Medical education

## Abstract

**Background:**

Formative assessment (FA) is becoming increasingly common in higher education, although the teaching practice of student-centred FA in medical curricula is still very limited. In addition, there is a lack of theoretical and pedagogical practice studies observing FA from medical students’ perspectives. The aim of this study is to explore and understand ways to improve student-centred FA, and to provide a practical framework for the future construction of an FA index system in medical curricula.

**Methods:**

This study used questionnaire data from undergraduate students in clinical medicine, preventive medicine, radiology, and nursing at a comprehensive university in China. The feelings of medical students upon receiving student-centred FA, assessment of faculty feedback, and satisfaction were analysed descriptively.

**Results:**

Of the 924 medical students surveyed, 37.1% had a general understanding of FA, 94.2% believed that the subject of teaching assessment was the teacher, 59% believed that teacher feedback on learning tasks was effective, and 36.3% received teacher feedback on learning tasks within one week. In addition, student satisfaction results show that students’ satisfaction with teacher feedback was 1.71 ± 0.747 points, and their satisfaction with learning tasks was 1.83 ± 0.826 points.

**Conclusion:**

Students as participants and collaborators in FA provide valid feedback for improving student-centred FA in terms of student cognition, empowered participation, and humanism. In addition, we suggest that medical educators avoid taking student satisfaction as a single indicator for measuring student-centred FA and to try to build an assessment index system of FA, to highlight the advantages of FA in medical curricula.

**Supplementary Information:**

The online version contains supplementary material available at 10.1186/s12909-023-04110-w.

## Background

During the COVID-19 pandemic, teaching models, assessments and feedback mechanisms in medical curricula were forced to adapt and make changes to minimize the negative impact of the epidemic on medical education [1, 2]. Although online curricula during the epidemic led to a greater diversity of content [3–5], medical curricula have strong professional characteristics. They not only spread medical knowledge, but also cultivate the logical thinking ability of medical students to find, analyse and solve problems. This makes the teaching assessment face many challenges [6, 7], a reduction in effective communication between students and faculty [8], a lack of depth and breadth in teaching models [9, 10], and a repetition of assessment components such as attendance, group reporting, and answering questions during face-to-face instruction [11]. Therefore, a new issue in the development of modern medical education is that medical educators focus on students, pay attention to their real feelings and feedback, encourage their participation in teaching assessment, and continuously improve teaching models and assessment methods [12, 13].

Student-centred includes a conceptual framework of three dimensions, namely cognitive (focus on student learning progress), agency (focus on student empowerment), and humanist (knowing students as individuals) [14, 15], which guided the design of this study. In student-centred teaching assessment design, cognition was reflected in the educator’s focus on student learning performance [16]; agency required the educator to consider how to enhance student engagement through power sharing [17]; and humanism is integrated throughout the teaching and learning process [17, 18], with the educator taking the initiative to understand students’ interests, desires, and needs. In other words, student-centred “power sharing” and “responsiveness to needs” are not separate, and teachers do not change their dominant position, but rather emphasize student agency, focusing on students’ learning experiences and meeting needs [14]. A recent student-centred study shows less attention to power sharing in East Asia than in other parts of the world [19], reminding us that student-centred teaching assessment needs to be validated in practice in a broader cultural educational context.

Formative assessment (FA) refers to the assessment in teachers and students systematically obtain evidence of students’ learning in the teaching process, promote students’ understanding of learning objectives, and support students to become learners and achieve learning objectives [20–23]. Teachers help students to establish a sense of ownership, continuously improve their own learning through teaching assessment, realize the value [24], and achieve assessment for learning. Modern medical education reform advocates for educators and researchers to support the realization of medical education goals, understanding and improving teaching assessment design [25, 26]. Although the assessment of medical curricula is mainly summative [27], focusing on students’ memory of knowledge, the purpose of FA is to focus on students, not only focusing on students’ scores, but also personal feedback and skills [28], especially the mastery of knowledge and skills. The assessment itself is used for learning and application to learning. FA is widely used in medical teaching practice and research, but not all assessments are effective. Effective FA feedback needs to consider how students participate and how teachers help students make effective feedback [29, 30].

Student satisfaction refers to the “subjective experience” of students in education and the perceived value of the learning experience [31, 32]. Guided by the concept of student-centred humanism, educators are particularly concerned about student satisfaction, and student satisfaction has been recognized as a valid indicator of instructional assessment in both theoretical and practical studies of teaching and learning [32]. Some studies have shown that improvement of teaching activities by teachers directly affects on student satisfaction [33]. However, well-designed teaching assessments may not obtain higher student satisfaction [34, 35]. This suggests a need for student-centred formative assessment and a deeper understanding of the value and role of student satisfaction, which has become a concern in medical education reform. We expect to understand medical students’ satisfaction after receiving student-centred FA and target the areas where FA needs to be changed. The quality of FA can be improved through scientific, appropriate, valid, and reliable methods [35]. At the same time, medical educators can benefit from feedback on students’ satisfaction and provide more appropriate teaching programs for independent learning [36].

Hence, this study selects medical students from a comprehensive university in China that has carried out medical curricula reform to observe and reflect on the content, methods, and feedback of teaching and learning assessments in medical curriculum through student-centred FA teaching practices. Based on the feedback regarding student satisfaction, we will also explore the shortcomings of FA as an indicator of student satisfaction and provide the best theoretical and practical framework for the design and implementation of FA in medicine or other disciplines.

## Methods

### Formative assessment design of medical curricula

The FA of medical curricula in a comprehensive university in China follows the principle of “teaching-assessment-feedback”. Starting from the feedback on teaching objectives, it investigates students’ views on the implementation of FA, matches with knowledge objectives, ability objectives and emotional value objectives, analyses whether the teaching objectives have been achieved, and teachers immediately give feedback their opinions to students. FA methods include topic discussion, classroom questions, and answers, classroom tests, reflection logs, mind maps, etc., focusing on quantification. Each assessment module has a different emphasis on the training of medical students’ knowledge and ability. Evaluators’ assessment methods include: mutual assessment between teachers and students, mutual assessment between students and students, and self-assessment by students and teacher assessment. The total score of each medical course is generally composed of the weighted and cumulative scores of each module of formative assessment, plus the final exam scores. Based on clinical medicine, FA has been extended horizontally to nursing, public health and preventive medicine and other specialties. It extends vertically from undergraduate education to postgraduate education.

In addition, the medical curriculum reform of this comprehensive university is student-centered, exploring the construction of a scientific and feasible FA index system, effectively evaluating teaching evaluation practices, and guiding teachers to cultivate students’ ability to reflect and learn independently. Therefore, from the perspective of students’ understanding, attitude and satisfaction to FA, enriching FA index system is worth discussing extensively.

### Participants

The study was designed as an investigative study. This comprehensive university in China have School of Clinical Medicine, School of Public Health, and School of Nursing. The undergraduate majors include clinical medicine, preventive medicine, radiology, and nursing. According to the research purpose, the research team has set the inclusion criteria for research objects, as follows: (1) undergraduate students receiving medical education, (2) undergraduate students who understand the research purpose; (3) undergraduate students who were voluntary to participate. Undergraduate students who did not receive medical course education, as well as undergraduate students who are conducting clinical practice, are excluded. The research team learned about the curriculum plan for the autumn semester of the academic year 2021–2022 in advance, and selected undergraduates majoring in clinical medicine, preventive medicine, radiology, and nursing from October 2021 to December 2021 in a comprehensive university in China for investigation.

The research team contacted instructors of medical curricula who distributed questionnaires in class through an online questionnaire platform (Questionnaire Star), and members of the research team answered the questions raised by the students when filling in the questionnaire. The completion of the questionnaire was voluntary for the students and informed consent was obtained from all participants. The study was approved by the ethics committee of the college where the research team leading member was based (Ethics Committee Approval Number: 2020092104).

### Questionnaire

The compilation of the questionnaire takes the student-centred conceptual framework of three dimensions as the theoretical basis of the research. In combination with the implementation background of FA in China’s medical education [15, 37], the questions designed are mainly divided into four parts: the basic demographic information of the respondents (gender, grade and major), medical students’ cognition of FA (degree of understanding, assessment scoring rules, main persons who completed the assessment, etc.), feedback (the effectiveness and timeliness of teachers’ feedback), satisfaction (FA method, content, tools informationization, scoring criteria, teacher feedback, and learning tasks). Among them, the satisfaction survey was scored with Likert’s five point scale, 1 to 5 was: very satisfied, satisfied, fair, dissatisfied or very dissatisfied. For the questionnaire in this study, Cronbach’s α = 0.976, KMO = 0.982, significance level *P* < 0.001, indicating the reliability and validity of the questionnaire are good. The questionnaire is listed in the Supporting materials 1.

### Statistical analysis

Statistical analysis was performed using SPSS 26.0, and all statistical tests were two-sided, with P < 0.05 considered statistically significant. Data descriptions of medical students’ demographic information, medical students’ cognition of FA, feedback were expressed as frequencies and percentages. The results of student satisfaction were analysed and expressed as Mean ± Standard Deviation. For the word frequency in the answer text of the open question, Excel was used to make a word cloud to analysis.

## Results

### Participant characteristics

In our study, there were 984 questionnaires distributed, 924 valid questionnaires were collected, and the efficiency was 93.9%. Of the 924 participants, 290 were male (31.4%), 634 were female (68.6%), fresh man was 302 (32.7%), sophomore was 295 (31.9%), junior was 210 (22.7%) and senior was 117 (12.7%). Clinical medicine 238 (25.8%), preventive medicine 240 (26.0%), radiation medicine 200 (21.6%) and nursing 246 (26.6%).

### Medical students’ cognition of formative assessment

**Fig. 1:**
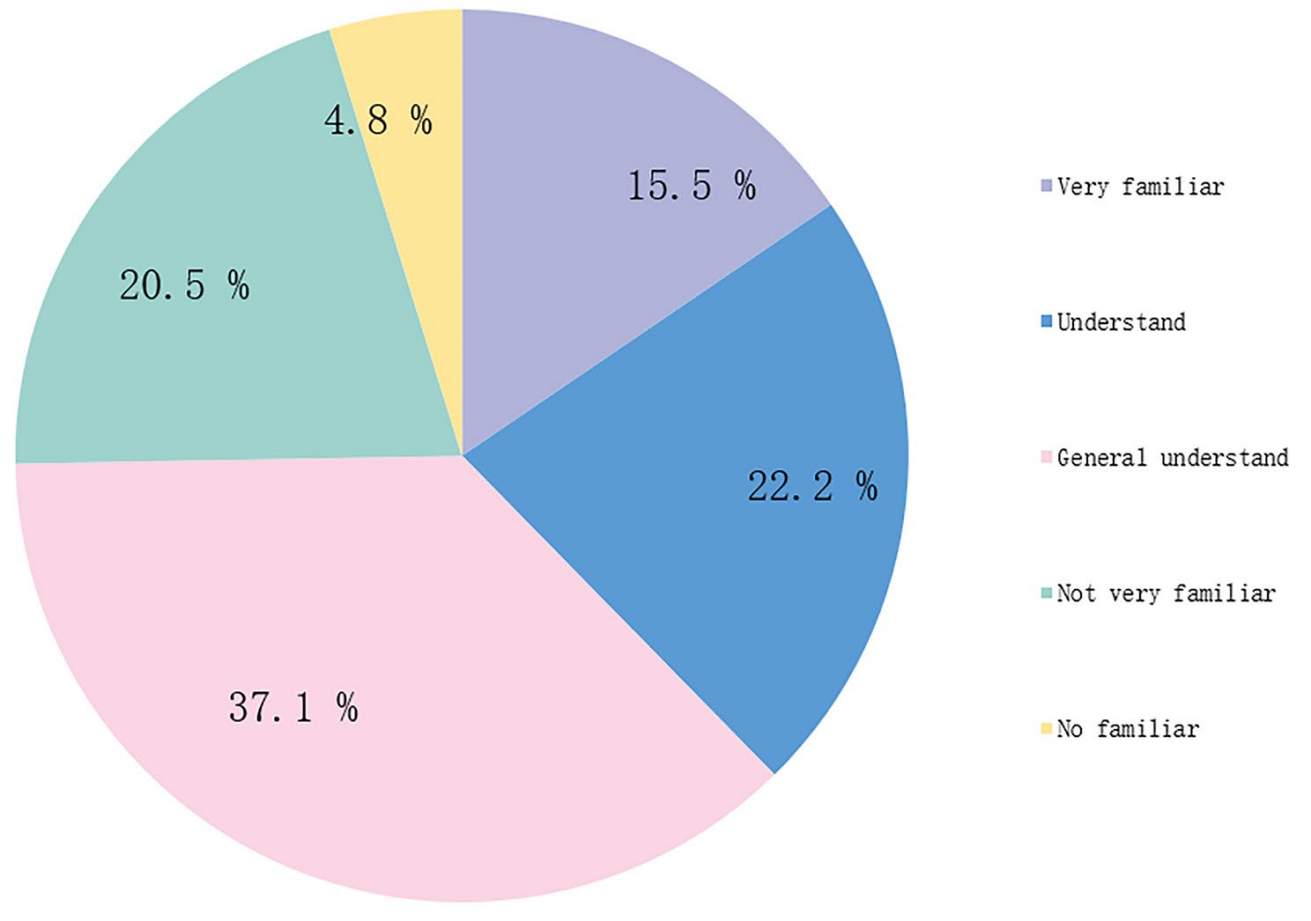
The degree of understanding of formative assessment by students

Students’ understanding of FA may help them participate in FA design, implementation and feedback. Before in-depth investigation, it is necessary to know the medical students’ understanding of FA. We through the question “How much do you know about formative assessment?”, preliminary understanding of medical students’ understanding of FA. Among the surveyed students, there were 143 (15.5%) very familiar, 205 (22.2%) understood, 343 (37.1%) general understood, 189 (20.5%) not very familiar, and 44 (4.8%) no familiar. (Fig. 1 and Supplemental Table 1).

### Students’ understanding of the scoring method of formative assessment

**Fig. 2:**
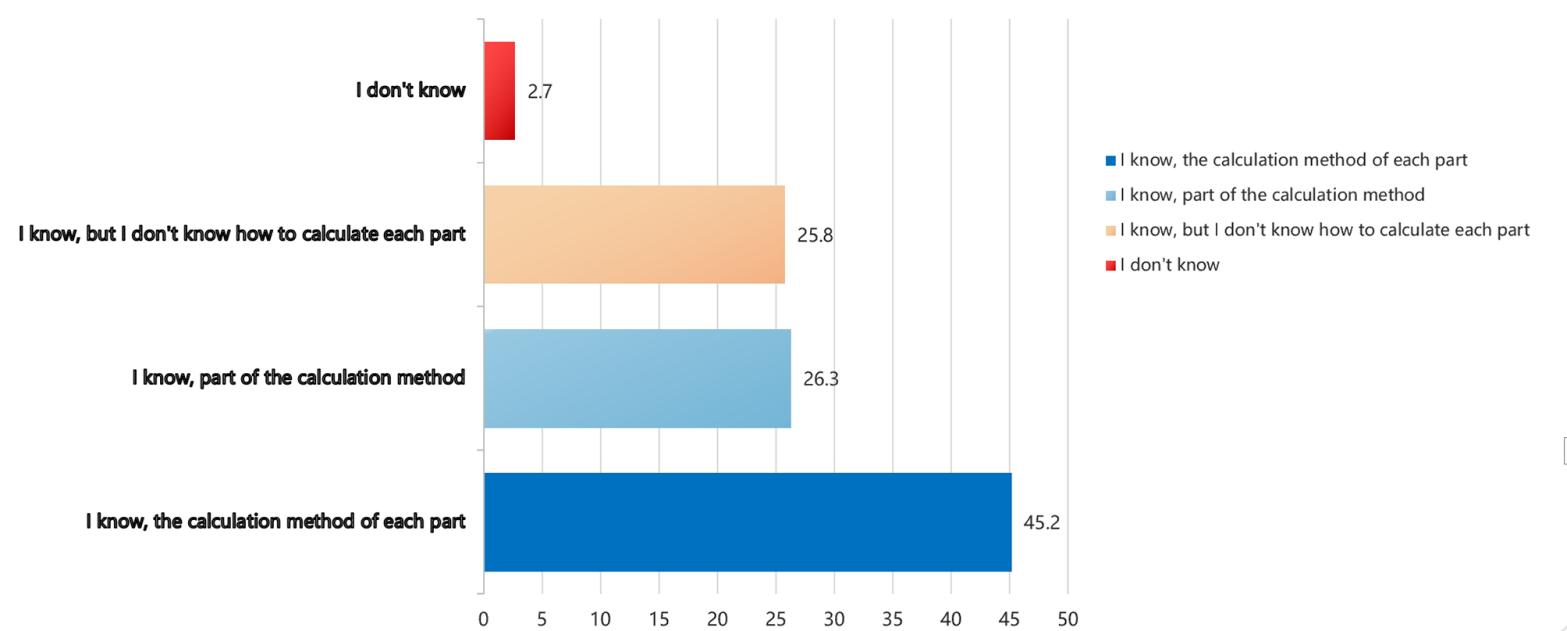
Students’ understanding of the scoring method of formative assessment

The content of FA is diverse, and the scoring method and weight of each method are different. In the survey of students, the question was “Do you know how to calculate the scores of each module of formative assessment?” They said they knew the calculation method of scores and the scores of each part, accounting for only 45.2% (Fig. 2 and Supplemental Table 2).

### Medical students’ assessment of formative assessment teachers’ feedback

The effective feedback of FA considers the participation of students and how teachers can help students provide effective feedback. When students receive teacher assessment tasks, they undertake these tasks to achieve learning. The timing of teacher feedback is essential for students to acquire knowledge, which is helpful in improving the efficiency of FA. In our research, the feedback survey of medical students on FA of teachers shows that 59.0% think “I get effective feedback”. From the timeliness of feedback, the number of medical students who received feedback from teachers was 335 (36.3%) within one week, 275 (29.8%) immediately, 112 (12.1%) at the end of the course, 98 (10.6%) the second day, 52 (5.6%) within one month and 52 (5.6%) no feedback (Table 1).

**Table 1:** Medical students’ cognition of formative assessment (N = 924)

Items	N	%
Have you received feedback from formative assessment?		
I get effective feedback	545	59.0
I get feedback, but it doesn’t help me much	108	11.7
I only get feedback a few times, but feedback helps me a lot	188	20.3
I only get feedback a few times, and feedback doesn’t help me much	61	6.6
Never get feedback	22	2.4
How long do you receive feedback from the teacher after completing the learning task?		
Immediately	275	29.8
The second day	98	10.6
Within one week	335	36.3
Within one month	52	5.6
After the course	112	12.1
No feedback	52	5.6

**Fig. 3:**
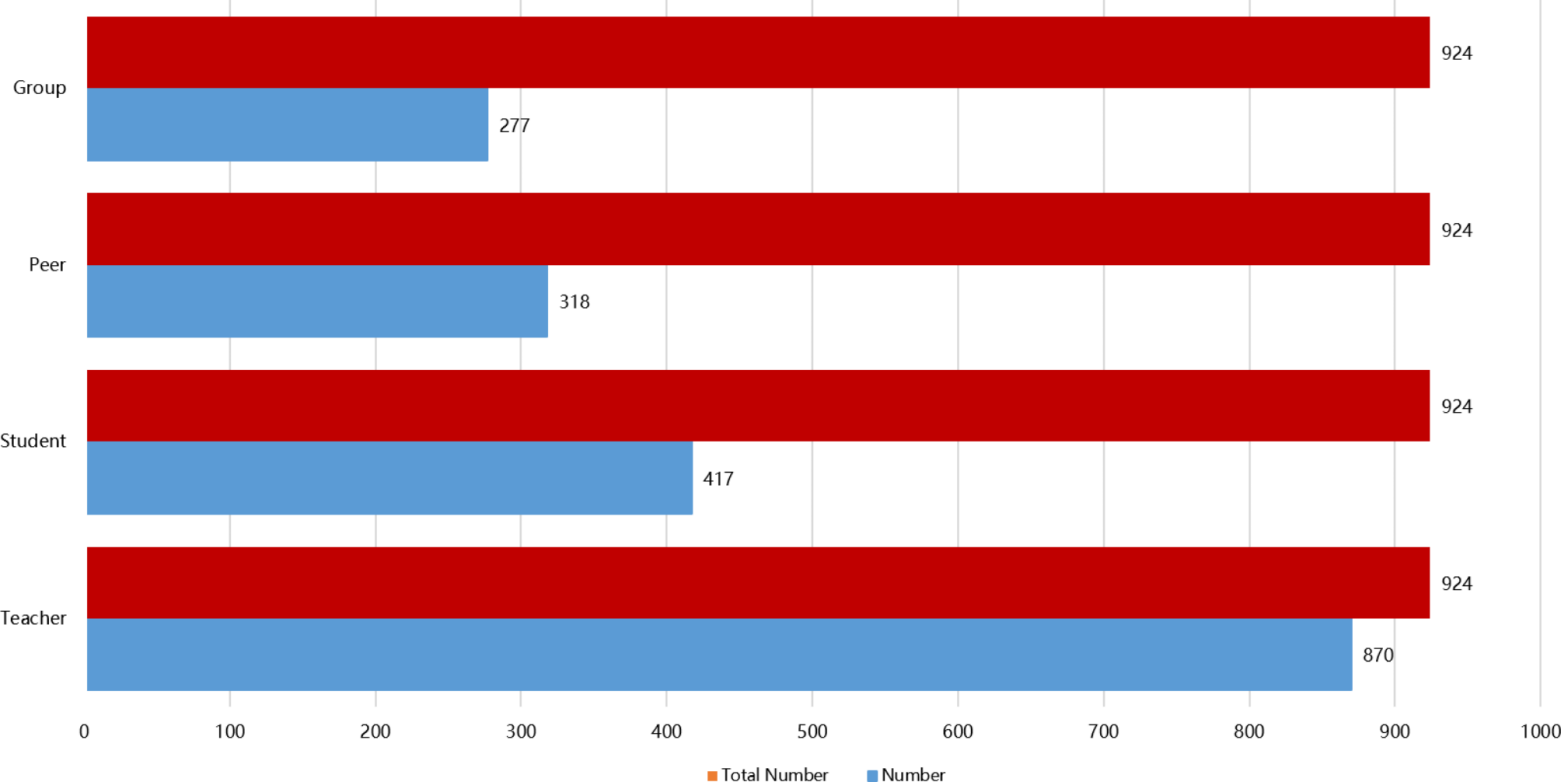
Students’ views on the main implementers of formative assessment

The subjects of FA are usually teachers and students, the objects of assessment may be teachers, students’ learning process, teachers’ teaching quality and students’ learning quality. In the multiple choice question “Who do you think is the subject of FA?“, the options we set include teacher, student, peer and group. According to the survey results, 94.2% (870/924) thought it was the teacher, 45.1% (417/924) thought it was the student, 34.4% (318/924) thought it was peer, and 30.0% (277/924) thought it was a group (Fig. 3 and Supplemental Table 3).

### Modules that medical students hope to add to the formative assessment of medical curricula

**Fig. 4:**
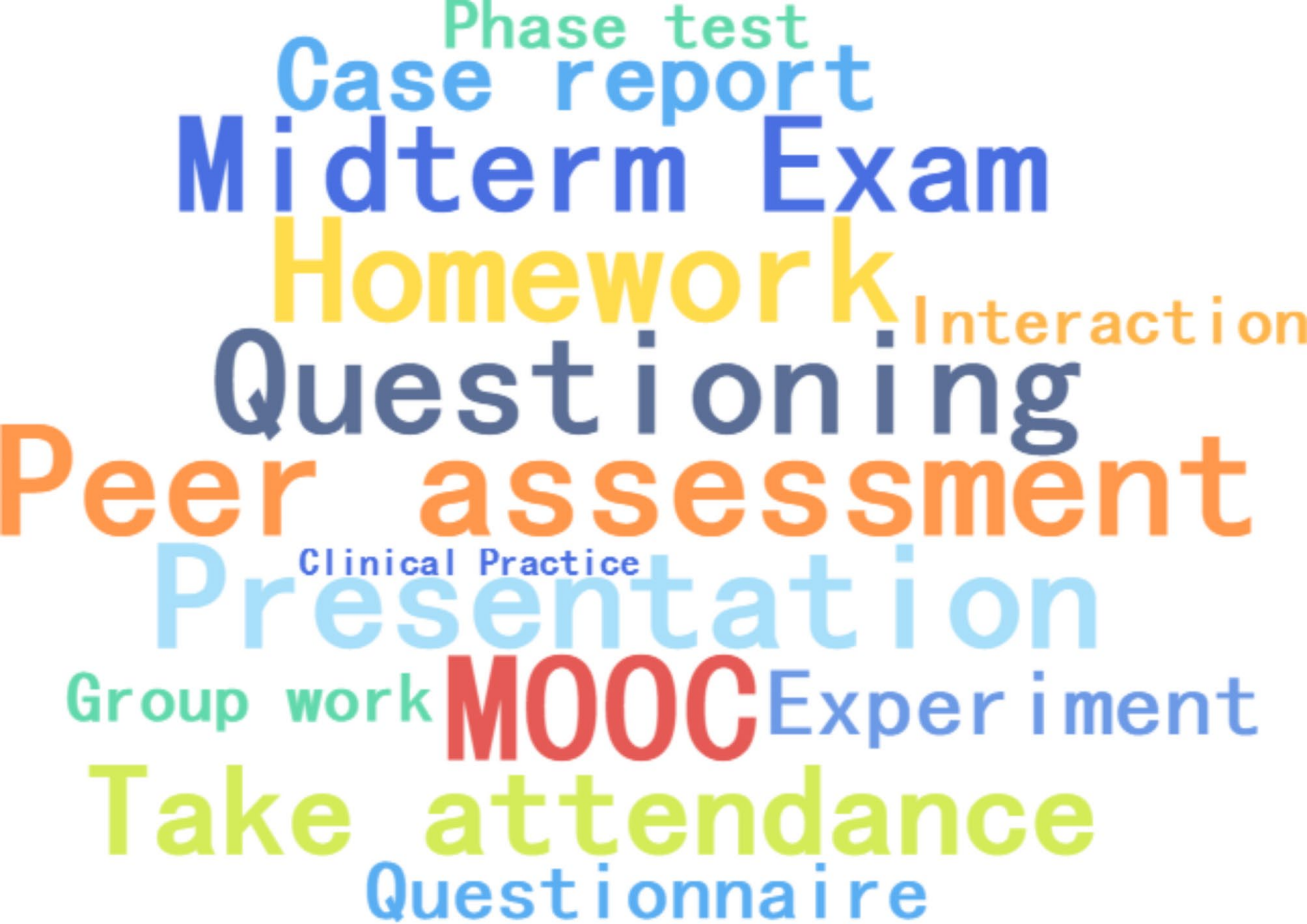
Formative assessment method preferred by medical students Note: The font size indicates the module frequency that medical students want to increase in formative assessment, the higher the frequency, the larger the font.

Open question “What formative assessment modules do you want to add in the future courses?“, The answer text is made into a word cloud, showing that the FA modules added by medical students in the future mainly include: peer assessment, presentation, questioning, take attendance, MOOC, etc. (Fig. 4 and Supplemental Table 4).

### Satisfaction of medical students with the formative assessment of medical curricula

**Fig. 5:**
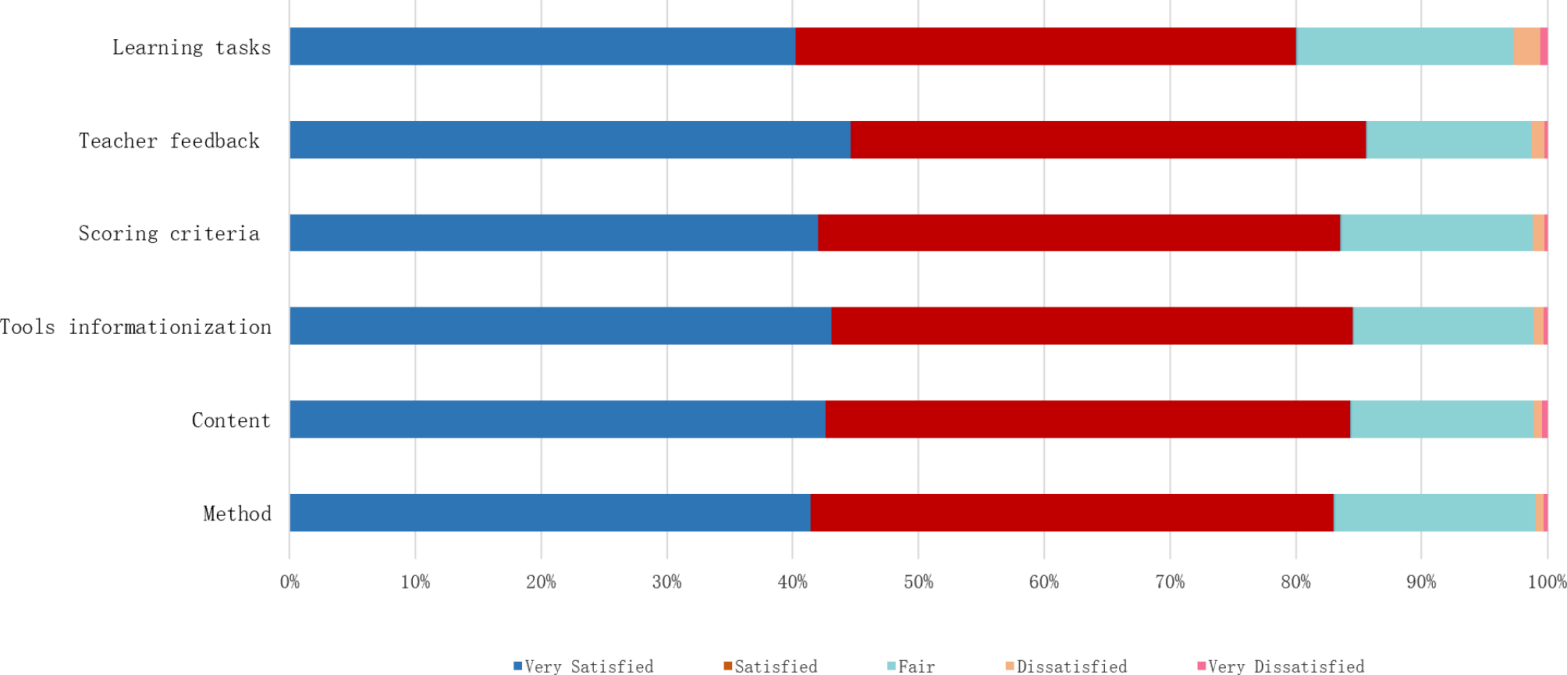
Satisfaction of medical students with formative assessment of medical courses

The results of medical students’ satisfaction with various items of FA can reflect students’ acceptance of FA. The higher the satisfaction of medical students, they may have better learning effect. When they have better learning effect, they will also have higher degree of satisfaction, so as to achieve the purpose of FA implementation. The satisfaction of medical students reflects the implementation effect of FA in terms of FA method, content, tools informationization, scoring criteria, teacher feedback, and learning tasks. In the investigation, we found that more than 40% of the students think they are very satisfied with the method, content, tools informationization, scoring criteria, teacher feedback, and learning tasks in medical curricula FA, the results of student satisfaction show that students’ satisfaction with teacher feedback was 1.71 ± 0.747 points. Their satisfaction with learning tasks was 1.83 ± 0.826 points (Table 2; Fig. 5).

**Table 2:** Satisfaction of medical students with the FA of medical curricula [N = 924, n(%)]

Items	Very Satisfied	Satisfied	Fair	Dissatisfied	Very Dissatisfied	Mean ± SD
Method	383 (41.5)	384 (41.6)	148 (16.0)	6 (0.6)	3(0.3)	1.77 ± 0.760
Content	394(42.6)	385(41.7)	135(14.6)	6(0.6)	4(0.4)	1.75 ± 0.757
Tools informationization	398(43.1)	383(41.5)	133(14.4)	7(0.8)	3(0.3)	1.74 ± 0.752
Scoring criteria	388(42.0)	384(41.6)	141(15.3)	9(1.0)	2(0.2)	1.76 ± 0.757
Teacher feedback	412(44.6)	379(41.0)	121(13.1)	10(1.1)	2(0.2)	1.71 ± 0.747
Learning tasks	372(40.3)	367(39.7)	160(17.3)	20(2.2)	5(0.5)	1.83 ± 0.826

## Discussion

To the best of our knowledge, this study was the first to survey undergraduate students covering clinical medicine, preventive medicine, public health, and nursing at a comprehensive Chinese university to analyse their perceptions, feedback, and satisfaction with implementation of student-centred FA in the medical curriculum. The results of this study indicate the necessity of medical students as subjects of FA, highlighting the concept of student-centred in three aspects: student cognition, empowered participation, and humanism. Of note is the value of student satisfaction as a measure of FA indicators.

Medical students have lower degree of understanding of FA, which hinders the efficiency and effectiveness of FA feedback. The survey results showed that 37.1% had a general understanding of FA, even though the instructor introduced students to the scoring of each task in the FA design during the first session of each course. In addition, only 45.2% of medical students clearly understood how each section of the FA was scored. This differs from the results of the FA methodology survey conducted with teachers, who were very clear about the methodology and purpose of FA [21, 23], while students were not. Possible reasons for this are that medical students are vague about the pedagogical goals of each FA task, are not yet clear about the attitudes, emotions, and values of FA, and are only passively working with teachers and completing assigned tasks. Future medical educators can pay attention to medical students’ cognitive development of teaching assessment and guide them to self-reflect and adjust their learning activities independently [38–41]. Suppose students lack the motivation to learn independently. In that case, the advantages of student-centred FA may be reduced, and teachers ask students ground to complete large, simple, and more repetitive tasks without thinking about what they are doing or learning, and without enjoying the authentic sense of accomplishment and satisfaction that comes from learning or completing tasks.

The power-sharing of student-centred FA, with teachers playing the roles of organizers, managers, and collaborators, attempts to encourage students to shift from passive acceptance of assessment to active participation in assessment and to guide students to become the subjects of FA [38, 42]. In our study, the scoring of FA, consisting of three components: student self-assessment, student mutual assessment, and teacher-student mutual assessment, consciously fostered the role of medical students as subjects in FA [43]. However, 94.2% of the medical students who participated in the survey perceived teaching and learning assessment as teacher-led, while 30% chose student-led. In contrast to the results of previous studies, traditional instructional assessment in medical curricula, which is dominated by instructor-led summative assessment, means that there is relatively little flexibility or opportunity to allow students to make decisions about their learning, thereby affecting their opportunities and motivation to participate in the assessment [24, 44]. Although medical student-centred FA received high levels of student satisfaction, medical students have a more ambiguous sense of themselves as assessment subjects. They remain stuck in an outdated notion that the teacher is the assessment in traditional teaching assessments [27, 41]. In addition, medical students reported in the open topic, their preference for using FA methods in the medical curriculum, and their preference for FA methods with an element of teacher-student interaction, reflecting their preference for classroom activities with immediate feedback. This differs from previous research findings in that traditional educational practices focus on teacher-led strategy implementation. In contrast, student-centred FA leaves decision-making about course activities to students, who are actively engaged by having a good experience in the interaction. Perhaps instructional assessment power-sharing with students is not the right way to assess student-centred instruction, but it represents a potentially effective way to do so.

Within the framework of student-centred theory, humanism focuses on understanding students’ aspirations, interests, and personalities and responding to them. The results of medical students’ satisfaction with each entry of FA in the survey showed that the mean score of the number of learning tasks reflected a low level of satisfaction, which is consistent with the results of previous FA satisfaction surveys [40, 45]. It is possible that students perceive that participating and completing the learning tasks of FA, with more time and effort, is still in a sense the same as traditional teaching assessment, passively completing the learning tasks assigned by teachers. Although student satisfaction is regarded as an important indicator to test teaching assessment, it does not mean that educators should cater to student satisfaction and reduce the amount and number of learning tasks [46]. At the same time, humanism advocates that the advantage of student satisfaction is to improve formative assessment by including students’ learning efficiency or teaching behavior, to promote students’ in-depth learning in FA. It is worth noting that some teachers may damage or reduce the FA standard to meet the students’ satisfaction, which affects the effectiveness of teaching evaluation. Avoid biased teaching evaluation results due to the limitations of student satisfaction [47], which may require the FA evaluation index system indicators to be diversified and complete, consider the dimensions of educators’ teaching behavior [48], students’ feedback and satisfaction with teaching evaluation [49], improve the single and one-sided defect of FA evaluation index system, and expand the positive impact of student satisfaction on student centered FA, promote teachers’ teaching and students’ learning.

Our study has several limitations that we recommend that future studies consider and address. First, we analyse medical students’ perspectives on the implementing of FA in the medical course, but this does not represent the perspectives of students in other disciplines. The medical course has the educational goal of developing medical personnel with professional, clinical competence and a strong sense of empathy, which is somewhat different from the educational goals of other disciplines, and future replication in other fields is needed to be able to generalize these findings. Second, the survey was completed voluntarily by students. Although the 93.9% response rate was reasonable, the lack of effective participation by some medical students did introduce a selection bias that should be considered a potential limitation. Finally, this study was cross-sectional, conducted during an epidemic, lacking in-depth follow-up, and lacking consideration of whether students’ perspectives have changed as the FA progressed, focusing on medical students’ overall competency over a more extended time and their updated recommendations for the FA.

## Conclusion

With the wide application of FA, students’ views and attitudes towards this kind of teaching assessment are becoming increasingly important. We identified the important value of students as participants and collaborators in FA. In addition, combined with student satisfaction, we suggest that medical educators avoid using student satisfaction as a single indicator to measure the teaching effectiveness of student-centred FA. In the future, they might also incorporate diverse assessment indicators to construct an assessment index system for FA to highlight the advantages of FA in medical curricula.

## Electronic supplementary material

Below is the link to the electronic supplementary material.

Supplementary Material 1

Supplementary Material 2

Supplementary Material 3

Supplementary Material 4

Supplementary Material 5

## Data Availability

The datasets used and/or analysed during the current study available from the corresponding author on reasonable request.
