# Supplementary material for: Reflection on the teaching of student-centred formative assessment in medical curricula: an investigation from the perspective of medical students
Source: BMC Med Educ. 2023 Mar 2;23:141. doi: 10.1186/s12909-023-04110-w (PMC9980864; doi:10.1186/s12909-023-04110-w)
Supplement: Supplementary file 1 — Supplementary Material 1 [file 12909_2023_4110_MOESM1_ESM.docx]

**Reflection on the teaching of student-centred formative assessment in medical curricula: an investigation from the perspective of medical students**

Tianjiao Ma, Yin Li, Hua Yuan, Feng Li, Shujuan Yang, Yongzhi Zhan, Jiannan Yao, Dongmei Mu

**From:** How much do you know about formative assessment?

**Supplemental Table 1.** Students' awareness of formative assessment (N=924)

| **Items** | **Number** | **Percentage (%)** |
| --- | --- | --- |
| Very familiar | 143 | 15.5 |
| Understood | 205 | 22.2 |
| General understood | 343 | 37.1 |
| Not very familiar | 189 | 20.5 |
| No familiar | 44 | 4.8 |
