# Supplementary material for: Reflection on the teaching of student-centred formative assessment in medical curricula: an investigation from the perspective of medical students
Source: BMC Med Educ. 2023 Mar 2;23:141. doi: 10.1186/s12909-023-04110-w (PMC9980864; doi:10.1186/s12909-023-04110-w)
Supplement: Supplementary file 2 — Supplementary Material 2 [file 12909_2023_4110_MOESM2_ESM.docx]

**Reflection on the teaching of student-centred formative assessment in medical curricula: an investigation from the perspective of medical students**

Tianjiao Ma, Yin Li, Hua Yuan, Feng Li, Shujuan Yang, Yongzhi Zhan, Jiannan Yao, Dongmei Mu

**From:** Do you know how to calculate the scores of each module of formative assessment?

**Supplemental Table 2.** Students' understanding of the scoring method of formative assessment (N=924)

| **Items** | **Number** | **Percentage (%)** |
| --- | --- | --- |
| I know, the calculation method of each part | 418 | 45.2 |
| I know, part of the calculation method | 243 | 26.3 |
| I know, but I don't know how to calculate each part | 238 | 25.8 |
| I don't know | 25 | 2.7 |
