# Supplementary material for: Reflection on the teaching of student-centred formative assessment in medical curricula: an investigation from the perspective of medical students
Source: BMC Med Educ. 2023 Mar 2;23:141. doi: 10.1186/s12909-023-04110-w (PMC9980864; doi:10.1186/s12909-023-04110-w)
Supplement: Supplementary file 3 — Supplementary Material 3 [file 12909_2023_4110_MOESM3_ESM.docx]

**Reflection on the teaching of student-centred formative assessment in medical curricula: an investigation from the perspective of medical students**

Tianjiao Ma, Yin Li, Hua Yuan, Feng Li, Shujuan Yang, Yongzhi Zhan, Jiannan Yao, Dongmei Mu

**Multiple choice question:** Who do you think is the main implementers of formative assessment?

**Supplemental Table 3.** Students' views on the main implementers of formative assessment

| **Categories** | **Number** | **Total Number** |
| --- | --- | --- |
| Teacher | 870 | 924 |
| Student | 417 | 924 |
| Peer | 318 | 924 |
| Group | 277 | 924 |
