# Supplementary material for: Reflection on the teaching of student-centred formative assessment in medical curricula: an investigation from the perspective of medical students
Source: BMC Med Educ. 2023 Mar 2;23:141. doi: 10.1186/s12909-023-04110-w (PMC9980864; doi:10.1186/s12909-023-04110-w)
Supplement: Supplementary file 4 — Supplementary Material 4 [file 12909_2023_4110_MOESM4_ESM.docx]

**Reflection on the teaching of student-centred formative assessment in medical curricula: an investigation from the perspective of medical students**

Tianjiao Ma, Yin Li, Hua Yuan, Feng Li, Shujuan Yang, Yongzhi Zhan, Jiannan Yao, Dongmei Mu

**From:** What other formative assessment methods do you hope to set up in the medical classroom?

**Supplemental Table 4. List of top frequency coded**

| **Frequency Coded** | **Frequency Coded（Chinese)** |
| --- | --- |
| Attendance roll call | 考勤点名 |
| Class discussion | 课堂讨论 |
| Classroom questioning | 课堂提问 |
| Group report | 小组汇报 |
| After class homework | 课后作业 |
| Literature report | 文献报告 |
| Mocha Video | 慕课视频 |
| experiment | 实验 |
| Practical Operation | 实操 |
| Final examination | 期末卷面考试 |
| Group presentation | 小组展示 |
| Flipped Classroom | 翻转课堂 |
| In class test | 随堂测试 |
| Teachers answer questions online | 教师线上答疑 |
| Students design experiments by themselves | 学生自己设计实验 |
| More interaction in class | 课堂多一些互动 |
| I hope the teacher can comment more on the homework | 希望老师多点评作业 |
| Group cooperation | 小组合作 |
| Chapter test question explanation | 章节测试题讲解 |
| Rush to answer questions | 问题抢答 |
| Students evaluate each other | 学生互相评价 |
| Anonymous questionnaire | 匿名问卷调查 |
| clinical practice | 临床实践 |
| Skill Operation | 技能操作 |
| Evaluation of experimental operation | 实验操作评价 |
| Real time classroom test | 实时课堂测试 |
| formative test | 阶段测试 |
| Case discussion | 病例讨论 |
| Online discussion | 线上讨论 |
