# Supplementary material for: Reflection on the teaching of student-centred formative assessment in medical curricula: an investigation from the perspective of medical students
Source: BMC Med Educ. 2023 Mar 2;23:141. doi: 10.1186/s12909-023-04110-w (PMC9980864; doi:10.1186/s12909-023-04110-w)
Supplement: Supplementary file 5 — Supplementary Material 5 [file 12909_2023_4110_MOESM5_ESM.docx]

**Reflection on the teaching of student-centred formative assessment in medical curricula: an investigation from the perspective of medical students**

**Supplement 1. Questionnaire translated into English**

Dear medical student,

The purpose of this survey is to understand the situation of formative assessment in medical courses. The survey results are only for research and are not used as a basis for judging academic achievements.

You are our best experts in the survey, your opinions are very valuable for research. Please fill it out carefully according to your real feelings.

Answering the questionnaire is voluntary. The answers will be treated confidentially and anonymity is guaranteed throughout the analysis and the report of the results.

You can answer the questionnaire by using the online format. Remember to send your answers by pressing the end command at the end of the questionnaire.

Thank you for your support and cooperation!

Yin Li

School of Public Health, Jilin University

e-mail: liyin@jlu.edu.cn

**Questionnaire: A Survey on the Application and Cognition of Medical Students on Medical Curriculum Formative Assessment**

The purpose of this questionnaire is to collect your true feelings and views about the formative assessment you received in the medical course, and provide us with suggestions for improving the teaching assessment.

By answering this questionnaire, you can help us develop our teaching practice!

**Background information**

College: _____________

Major: _____________

Student number: _____________

Name: _____________

Gender:

① Male

② Female

Grade:

① Fresh man

② Sophomore

③ Junior

④ Senior

**Cognition of medical students on formative evaluation of courses**

1. How much do you know about formative assessment

① Very familiar

② Understood

③ General understood

④ Not very familiar

⑤ No familiar

**Formative evaluation feedback**

1. Have you received feedback from formative assessment? (such as: homework has been annotated; PPT report has been commented; teachers explain problems; provide suggestions for improvement of homework, etc.)

① I get effective feedback

② I get feedback, but it doesn't help me much

③ I only get feedback a few times, but feedback helps me a lot

④ I only get feedback a few times, and feedback doesn't help me much

⑤ Never get feedback

2. How long do you get teaching feedback after completing the learning activity

① Immediately

② The second day

③ Within one week

④ Within one month

⑤ After the course

⑥ No feedback

**Application of formative assessment of medical courses**

1. Among the medical courses you have studied, What percentage of formative assessment is used.

① All courses are carried out (100%)

② Some courses (less than 50%)

③ No courses (0)

2. Do you know how to calculate the scores of each module of formative assessment

① I know, the calculation method of each part

②I know, part of the calculation method

③ I know, but I don't know the score of each part

④ I don't know

3. (Multiple choice questions) Who do you think is the main implementers of formative assessment

① Student

② Teacher

③ Peer

④ Group

4. (Open question) What other formative assessment methods do you hope to set up in the medical classroom

**Satisfaction of medical students with formative assessment of medical courses**

1. How satisfied are you with the assessment method of formative assessment of medical courses

① Very satisfied

② Satisfied 

③ Generally satisfied

④ Unsatisfactory

⑤ Very dissatisfied

2.How satisfied are you with the assessment content of formative assessment of medical courses

① Very satisfied

② Satisfied 

③ Generally satisfied

④ Unsatisfactory

⑤ Very dissatisfied

3. How satisfied are you with the assessment tools informationization of formative assessment of medical courses

① Very satisfied

② Satisfied 

③ Generally satisfied

④ Unsatisfactory

⑤ Very dissatisfied

4. How satisfied are you with the assessment scoring criteria of formative assessment of medical courses

① Very satisfied

② Satisfied 

③ Generally satisfied

④ Unsatisfactory

⑤ Very dissatisfied

1. How satisfied are you with the assessment teacher feedback of formative assessment of medical courses

① Very satisfied

② Satisfied 

③ Generally satisfied

④ Unsatisfactory

⑤ Very dissatisfied

6.How satisfied are you with the assessment learning tasks of formative assessment of medical courses

① Very satisfied

② Satisfied 

③ Generally satisfied

④ Unsatisfactory

⑤ Very dissatisfied

Thank you for answering the questionnaire!

Inquiries concerning the research and the questionnaire:

Yin Li, e-mail: liyin@jlu.edu.cn

Tianjiao Ma, e-mail: matianjiao216@jlu.edu.cn

Remember to press the ‘send’ button at the end of the webpage.
